# Poster Session II - A261 TREATMENT OF CROHN’S DISEASE-ASSOCIATED GASTRIC OUTLET OBSTRUCTION: A SCOPING REVIEW

**DOI:** 10.1093/jcag/gwaf042.261

**Published:** 2026-02-13

**Authors:** A K Powar, A Hosseinzadeh, B Nguyen

**Affiliations:** The University of British Columbia, Vancouver, BC, Canada; The University of British Columbia, Vancouver, BC, Canada; The University of British Columbia, Vancouver, BC, Canada

## Abstract

**Background:**

Gastric outlet obstruction (GOO) is a rare complication of Crohn’s disease (CD), resulting from stricturing gastroduodenal disease. Management is complex, with medical, endoscopic, and surgical options reported.

**Aims:**

To synthesize evidence on the management of CD-associated GOO and compare treatment success rates.

**Methods:**

Following PRISMA guidelines, MEDLINE, PubMed, and Embase were searched for studies describing GOO secondary to CD. Case reports, case series, and observational studies reporting treatment outcomes were included. Non-English studies were excluded.

**Results:**

Of 282 studies screened, 57 studies comprising 230 patients were included. We identified 295 treatment trials for CD–related GOO. Success was defined as complete resolution of symptoms and absence of recurrence at the end of the follow-up period. Outcomes for treatment regimens are summarized in Table 1 and Figure 1. Surgery achieved the highest success (94%, n = 80 for surgery alone; 100%, n = 7 for surgery plus medical therapy), and was offered as first-line therapy in 41% of cases. Endoscopic balloon dilation (EBD) achieved 65% success alone (n = 133) and 75% when combined with medical therapy (n = 20). Medical therapy alone with disease-modifying drugs was less effective (50%, n = 26 for single agents; 44%, n = 16 for combination regimens), while supportive therapy alone (n = 13) did not achieve any resolution. Considering each modality independently of the regimen, success rates were highest for surgery (94%), followed by TNFα inhibitor biologics (68%) and EBD (66%). EBD alone was the most common first-line approach resulting in complete resolution of GOO.

**Conclusions:**

Surgery provides the most durable resolution of CD-related GOO. However, EBD is frequently effective and less invasive, making it a reasonable initial option. Medical therapy alone is less effective, though regimens including TNFα inhibitor biologics may have a role. This scoping review is the first to synthesize treatment strategies for this uncommon manifestation of CD.

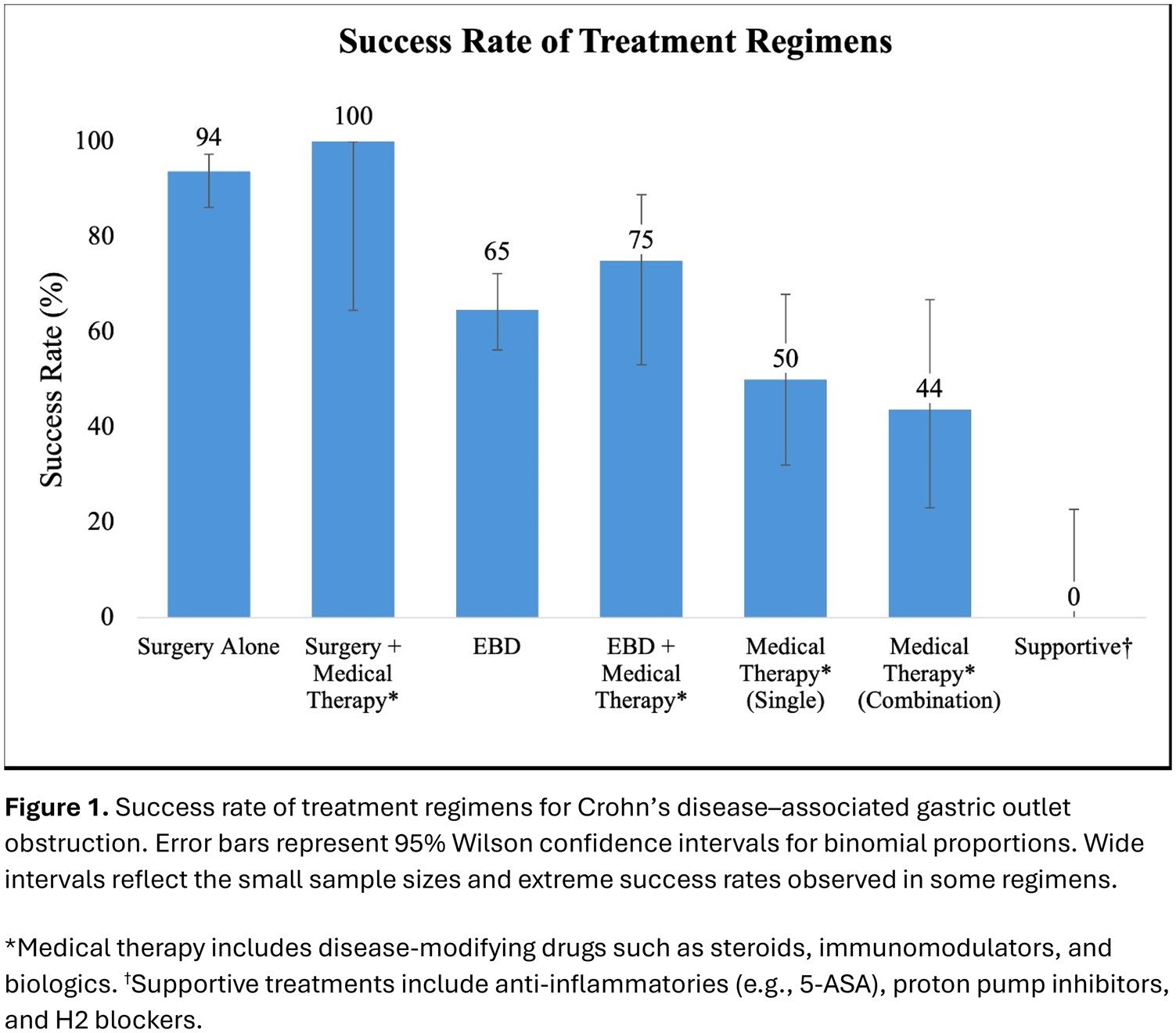

**Funding Agencies:**

None

**Table 1:** A261 Outcomes of treatment regimens for Crohn’s disease–associated gastric outlet obstruction

Treatment Regimen	Complete Resolution (n)	Incomplete Resolution (n)	Success Rate (%)	CD Diagnosis Established Before Treatment (%)	First-Line Use Within Treatment Regimen (%)	Proportion of All First Line Treatments (%)
Surgery Alone	75	5	94	90	41	16
Surgery + Medical Therapy^*^	7	0	100	57	43	1
EBD	86	47	65	98	95	60
EBD + Medical Therapy^*^	15	5	75	90	40	4
Medical Therapy^*^ (Single)	13	13	50	96	69	9
Medical Therapy^*^ (Combination)	7	9	44	94	50	4
Supportive†	0	13	0	38	92	6

* Medical therapy includes disease-modifying drugs such as steroids, immunomodulators, and biologics.

† Supportive treatments include anti-inflammatories (e.g., 5-ASA), proton pump inhibitors, and H2 blockers.

